# Resistance and tolerance reactions of winter wheat lines to *Heterodera filipjevi* in Turkey

**DOI:** 10.21307/jofnem-2019-031

**Published:** 2019-06-10

**Authors:** Abdelfattah A. Dababat

**Affiliations:** 1 International Maize and Wheat Improvement Center (CIMMYT), P.K. 39 Emek, 06511, Ankara, Turkey

**Keywords:** Breeding, Cyst nematode, *Heterodera filipjevi*, Host reaction, Resistant, Yield losses, Wheat

## Abstract

Nematodes attack cereal crops resulting in significant yield losses, estimated at 10%. The plant parasitic nematodes of the genus *Heterodera* attack cereals, particularly wheat, causing costly financial losses due to impact on yield. The soil borne pathogens program at the International Maize and Wheat Improvement Center (CIMMYT) in Turkey has focused on screening wheat germplasm to identify sources of *Heterodera* resistant varieties for almost 20 years. The aim of this current study was to validate the finding that resistant lines demonstrate resistant reactions under controlled conditions and to test whether they present tolerant reactions when challenged with cyst nematodes under two different locations in field conditions. The results of this study, including the check lines, indicated that 27 and 28 lines maintained their reactions to *H. filipjevi* in Eskisehir and Yozgat field, respectively, and 23 lines were the same in both locations. In terms of tolerance, 3 and 13 lines proven to be tolerant and moderately tolerant to *H. filipjevi* in Yozgat field. In Eskisehir field, 13 and 14 lines were tolerant and moderately tolerant. In both locations, L7 showed tolerance reaction, although it was susceptible. The majority of the resistant germplasm (60%, 14 lines) of screened lines from the Turkey CIMMYT–ICARDA (TCI) nursery were found to be resistant to both *H. filipjevi* populations including L1, L3, L6, L15, L21, L26, and L34, whereas 17% (four lines) from the USA had the same reaction. L32 showed a high level of resistance and tolerance in both locations and could prove to be promising lines in the breeding programs. The International Winter Wheat Improvement Program (IWWIP) formerly used these resistant lines in the crossing block and subsequently distributed them to more than 150 international collaborators. Regression analysis revealed a negative correlation between yield and RF of *H. filipjevi* in both nematode populations, which describes the negative impact of this pest on winter wheat. The results of this study are very important for breeding programs especially for the IWWIP, a joint program between the Turkish Ministry of Agriculture and Forestry, CIMMYT, and the International Centre for Agricultural Research in the Dry Areas (ICARDA).

Wheat (*Triticum aestivum* L.) is a vital staple food crop. An estimated 750 million tons (MT) of the grain was grown on more than 220 million hectares (Mha) worldwide in 2017 (Wuletaw et al., 2016; Dababat and Hendrika, 2018). Turkey contributed more than 22 MT over 7.77 Mha in 2013 (FAO, 2018). The world’s population is expected to reach 9 to 10 billion in 2050; thus, based on population growth, cereal production must be increased by 50% by 2030 to meet demand (Alexandratos and Bruinsma, 2012).

The average area (in Mha) of production (in MT) and yield (tonnes/ha) of wheat from 2008 to 2012 by country in the Central West Asia and North Africa (CWANA) region is depicted as reviewed by Wuletaw et al. (2016). Their research indicates that low productivity levels of wheat in the CWANA region is due to abiotic stresses (drought, cold, heat, and salinity) and biotic stresses (stripe rust, leaf rust, stem rust, root rots, Russian wheat aphid, barley yellow dwarf virus, sunn pest, and Hessian fly). Recently, Wuletaw et al. (2016) also reviewed wheat production in Sub-Saharan Africa under a changing climate.

Cereal cyst nematode (*Heterodera avenae* complex; CCN) is found globally and leads to significant economic yield losses, particularly in areas where dryland and cereal monoculture systems are practiced (Nicol et al., 2003; Dababat et al., 2015). After hatching, second-stage juvenile (J2) cyst nematodes start attacking the roots of the plant and begin feeding. The J2s penetrate the root system and after mating, females produce several 100 eggs inside their bodies. The eggs grow, transforming the female into an oblate spheroid. When the host plant begins to die, the cuticle hardens and becomes a cyst, which stores the eggs until the following growing season when they hatch (Dababat et al., 2015). CCN accelerates synergistic negative effects when combined with other biotic and abiotic factors, such as drought and fungal pathogens (Nicol and Ortiz-Monasterio, 2004; Nicol et al., 2006; Dababat et al., 2018). Damage caused by CCN has been reported from different parts of the world and is estimated to have caused grain losses at rates of 20, 90, 50, and 24% in Pakistan, Saudi Arabia, Australia, and the USA, respectively (Nicol, 2002; Dababat and Hendrika, 2018). CCN (mainly *H. avenae*) is calculated by Barker et al. (1998) to have caused losses of about $78 billion around the globe. Worldwide, cereal production losses are estimated at 10% due to plant-feeding nematodes (Whitehead, 1998).

So far, several attempts have been made to control CCN, including through chemicals (Dababat et al., 2014, 2015), crop rotation (Smiley et al., 2008), genetic resistance (Dababat et al., 2014, 2015, 2016), and biological control (Ashrafi et al., 2018). Genetic resistance is the most reliable control option as it is cheap, easy to use once identified, and environmentally friendly. Ideally, resistance (the ability of the plant to inhibit nematodes multiplication) should be combined with tolerance (the plant’s ability to withstand infection and produce yields despite the CCN attack) (Smiley et al., 2004).

However, on a global scale, there are very few programs working to breed for nematode resistance in wheat. Plant parasitic nematodes are considered to be one of the leading destructive diseases attacking wheat and cause significant yield losses (Dababat and Hendrika, 2018). As yield loss to nematodes presents a clear obstacle to achieving the goal of increased cereal production, a targeted breeding program will be necessary to discover new sources of nematode resistance in wheat.

The soil-borne pathogen program at CIMMYT Turkey annually receives about 1,000 accessions of wheat from the CIMMYT Mexico spring wheat program and IWWIP. They are screened under growth room, greenhouse and field conditions at various locations in Turkey. Cultivars are also screened for multiple disease resistances, such as resistance to different species of root lesion nematodes (e.g. *Pratylenchus thornei* and *P. neglectus*), other cyst-forming nematodes (e.g. *H. avenae, H. filipjevi*, and *H. latipons*) and two *Fusarium* species (*F. culmorum* and *F. pseudograminearum*). The most resistant lines are then distributed to international collaborators for use in their breeding programs.

However, the habits and nature of CCN is complex due to the existence of pathotypes, which require long-term investment strategies. This is vital to ensure that a selected resistant wheat line can target as many different pathotypes as possible in addition to other *Heterodera* species. Therefore, the aims of this study were: (i) to screen winter wheat germplasm provided by IWWIP against the cereal cyst nematode *H. filipjevi*, (ii) validate the resistance of those lines under the infested fields with *H. filipjevi*, and (iii) study and compare the tolerance reaction of lines under field conditions with the proven resistant ones under controlled conditions.

## Materials and methods

### Germplasm selection

During the annual screening of the winter wheat germplasm provided by IWWIP, a group of 31 entries showed promising resistance potential to *H. filipjevi* under growth room conditions at the Transitional Zone Agricultural Research Institute (TZARI) in Eskisehir, Turkey and were selected for this study. Four standard check lines: Katya (MR), Sonmez (MR), Kutluk (S), and Bezostaya (S), being well-recognized for their resistant/susceptible reactions to *H. filipjevi*, were also included as known controls (Table 1) (Dababat et al., 2015).

**Table 1.** The list of the winter wheat germplasm used in the field studies plus the four check lines with their resistant and tolerant reactions in both fields.

Line	NURS		C-NAME	CID	OC	ACC NO	R – Yoz	R – Esk
1	13CAND-IWWYT-SA	102	ORKINOS-1/4/JING411//PLK70/LIRA/3/GUN91	TCI041519	TCI	110150	R, T	R, MIT
2	13CAND-IWWYT-SA	105	GUN91/4/SNI//CAR422/ANA/3/KAUZ*2/TRAP//KAUZ/5/MERCAN-1	TCI032074	TCI	110044	R, MIT	R, MT
3	13CAND-IWWYT-SA	113	SABALAN/3/PVN/BOW//LIVA/4/MERCAN-2/5/TX96V2427	TCI032546	TCI	110187	R, MIT	R, MT
4	13CAND-IWWYT-SA	115	GUN91/3/CROC_1/AE.SQUARROSA (205)//KAUZ/4/IZGI	TCI032143	TCI	110116	S, MIT	R, MT
5	13CAND-IWWYT-SA	127	PMF/MAYA//YACO/3/CO693591/CTK/4/F1-1S-1/CHISHOLM	TCI02-142	TCI	100518	R, MT	R, MT
6	13CAND-IWWYT-SA	138	DORADE-5//KS82117/MLT	TCI-02-88	TCI	090437	R, IT	R, T
7	C21FAWWON-INT	27	OK08413		USA-OK	110575	S, T	S, T
8	C21FAWWON-TCI	8	KATE A-1	Hebros/Bez-1		950590	R, MIT	R, T
9	C21FAWWON-TCI	10	SERI.1B//KAUZ/HEVO/3/AMAD/4/TAM111/5/T67/JGR //ARLIN	OCW03S238T	USA-OK-TCI	110380	R, MT	R, T
10	C21FAWWON-TCI	17	KARAHAN-99	C126-15/COFN“S”/3/N10B11/P14//SEL101/4/KRC	YE 2957-4E-1E-1E-0E	920007	MS, IT	R, MT
11	C21FAWWON-TCI	29	GRISET-4/3/ID#840335//PIN39/PEW/4/LILIA BG/GT	TCI032267		110249	R, IT	R, MIT
12	C21FAWWON-TCI	36	T67/X84W063-9-45//KARL92/3/GUN91/MNCH/4/SAULESKU #44/TR810200	TCI032527	TCI	110377	R, MT	S, T
13	C21FAWWON-TCI	55	JCAM/EMU//DOVE/3/JGR/4/THK/5/BOEMA	TCI031188	TCI	110160	R, MT	R, T
14	C21FAWWON-TCI	60	KIRGIZ95/8/SABUF/7/ALTAR 84/AE.SQUARROSA (224)//YACO/6/CROC_1/AE.SQUARROSA (205)/5/BR12*3/4/IAS55*4/CI14123/3/IAS55*4/EG,AUS//IAS55*4/ALD/9/MEZGIT-4	TCI032082	TCI	110174	R, MT	S, MT
15	C21FAWWON-TCI	62	KS920709-B-5-1-1/4/CHAM6//1D13.1/MLT/3/SHI4414/CROW	TCI031396	TCI	110347	R, MT	R, MT
16	14Sbpcl	37	Kutluk				S, MIT	S, IT
17	C21FAWWON-TCI	68	GUN91/3/CROC_1/AE.SQUARROSA (205)//KAUZ/4/IZGI	TCI032143	TCI	110116	R, MIT	R, IT
18	C21FAWWON-TCI	75	GEREK			950497	R, MT	R, IT
19	12CBWF	68	HBA142A/HBZ621A//ABILENE/3/CAMPION/4/F6038W12.1	TCI012144	TCI	090350	R, MT	R, T
20	12CBWF	212	PYN/BAU/3/KAUZ//KAUZ/STAR	CMSW01WM00586S	MX-TCI	090493	S, MIT	R, T
21	C20FAWWON-TCI	6	ATTILA/2*PASTOR//YUMAI 29	OCW02S567S	OK-TCI	100064	R, MT	R, T
22	C20FAWWON-TCI	7	ATTILA/2*PASTOR//YUMAI 29	OCW02S567S	OK-TCI	100065	R, MT	R, MT
23	C20FAWWON-TCI	10	PFAU/WEAVER/3/MASON/JGR//PECOS	OCW02S369S	OK-TCI	100068	R, MIT	S, MT
24	C20FAWWON-TCI	20	TAM200/HBB313E//2158 (OK98697)/5/SITE/MO/4/NAC/TH.AC//3*PVN/3/MIRLO/BUC/6/JGR/CUSTER//JGR* (OK0062278)	OCW02S155T	OK-TCI	100120	S, MIT	S, MT
25	C20FAWWON-TCI	32	KAMBARA1/KALYOZ-17	TC1021034	TCI	100365	R, MT	R, MIT
26	C20FAWWON-TCI	108	NALIM-3/ZHETISU/5/Sonmez=ES98KE14=NALIM-4=BEZ//BEZ/TVR/3/KREMENA/LOV29/4/KATIA1	TCI022272	TCI	100213	R, IT	R, MT
27	C20FAWWON-TCI	117	SONMEZ				R, IT	R, MT
28	C20FAWWON-INT	334	cv. Rodina/Ae.speltoides (10 kR)	179/98w	RUS	101519	R, IT	R,T
29	C20FAWWON-INT	343	OR2071029		OR-USA	100988	R, IT	R,T
30	C20FAWWON-INT	372	Passarinho//Vee/Nac	1-NS 1590	Iran-Karadj	110479	R, MT	R, MT
31	C20FAWWON-INT	391	AWD99*5725/FL9547	ARS09-040	US-NC	110509	R, MT	S, T
32	C20FAWWON-INT	403	NC00-14622/2137	ARS09-382	US-NC	110531	R, T	R, T
33	C20FAWWON-INT	408	GA951079-3-5/TX99D4628	ARS09-556	US-NC	110545	R, IT	R, MIT
34	C20FAWWON-INT	532	4WON-IR-257/5/YMH/HYS//HYS/TUR3055/3/DGA/4/VPM/MOS	TCI-02-80	TCI	090082	R, MIT	R, MT
35			BEZOSTAYA				S, IT	S, IT

Note: Ent, entry; Nurs, nursery; C-Name, cross name; CID, cross identification; OC, origin country; Acc No, accession number; R - Yoz, resistant reaction; Yoz, Yozgat; Esk, Eskisehir; TCI, Turkey CIMMYT -ICARDA; MX, Mexico; US-NC, United State - New Jersey; USA-OK, USA - Oklahoma; R, resistant; S, susceptible; MS, moderately susceptible; MT, moderately tolerant; T, tolerant; MIT, moderately intolerant; IT, intolerant.

### Experimental sites

All genotypes, including the checks, were tested in two different fields: one at the ILCI Cicekdağı Agricultural Enterprise (ICAE) in Yozgat, Turkey (Latitude 39.63806; Longitude 34.46722) and one at TZARI in Eskisehir (Latitude 39.76670; Longitude 30.40518). The trials in each of these two locations were conducted during the 2014 to 2015 season and repeated in 2015 to 2016. The ICAE site is identified as historically infested with substantial populations of *H. filipjevi* based on the molecular identification (Cui et al., 2017), while the TZARI site was artificially inoculated as of 2012. The two sites are located in the Central Anatolian Plateau, which is characterized by medium rainfall. Precipitation totaled 274 mm and 318 mm for Yozgat and Eskisehir in the 2014 to 2015 crop years, respectively. Most (169.2 mm and 232 mm) fell between April and July in Yozgat and Eskisehir, respectively (TSMS, 2018). In 2015 to 2016, overall, 294 mm and 348 mm precipitation fell in Eskisehir, and 176 mm and 250 mm fell between April and July, respectively (TSMS, 2018). The mean high temperatures for July and August were 28.6°C and 26.9°C in Yozgat, and 26.2°C and 24.5°C in Eskisehir for the 2014 to 2015 and 2015 to 2016 growing seasons, respectively (TSMS, 2018). Generally, soil at ICAE and TZARI is characterized by shallow, sandy clay loam with a plant-limiting hardpan layer at a depth of 20 cm.

### Field experiments

Trials were planted in October for both locations and growing seasons, with each entry sown in a plot (1 m wide × 2 m long) consisting of four rows with 20 cm between rows in the TZARI field, whereas plots in ICEA were 5 m long × 1.2 m wide consisting of six rows with 20 cm between rows. Seeds were sown at a rate of 550 to 600 seeds per square meter. A randomized complete block design was used in both locations with three replicates. Trials were fertilized at sowing time with 10–10–10–5 NPKS at 200 kg/ha. Bronate® herbicide (MCPA + bromoxynil) was applied at 470 ml/ha to control broadleaf weeds. Trials were terminated in July. At harvest, spikes from each plot were manually harvested using a sickle and threshed with a small harvester at TZARI and by experimental harvesting machine in ICEA. Grain weight per plot was weighed and recorded.

### Nematode sampling

Six soil subsamples of 250 cm^3^ each were taken per plot in a zigzag pattern at the beginning of the growing seasons to assess nematode initial populations (*P*_i_). Samples were taken from around seed rows at 20 cm depth using a soil auger (2.5 cm diameter). Extraction of the cysts from the soil was performed as per Dababat et al. (2014) following Fenwick’s (1940) can floatation methods for cyst extraction. These trials were replicated for data validation. At maturity or at harvest (early July), similar soil sampling and extraction methods were performed to determine nematode final population density (*P*_f_) per plot by taking soil samples from the root niche. The nematode *P*_i_ and *P*_f_ were used to calculate the nematode reproduction factor (RF) based on equation: RF = *P*_f_/*P*_i_.

An accession’s resistant reaction was classified into one of five distinctive groups based on the reproduction factor (RF): resistant (R) = RF equal or less than 1; moderately resistant (MR) = RF between 1 and 2, a few more cysts than in a resistant check; moderately susceptible (MS) = RF between 2 and 3, distinctly more cysts than in a resistant check, but less than in the susceptible check; susceptible (S) = RF between 3 and 4, more cysts than in the susceptible check; and highly susceptible (HS) = RF more than 4, cyst number higher than in the susceptible check (Dababat et al., 2016) and taking into the account the reaction of the known check lines used in the study. The accession’s tolerance reaction was classified into four groups based on the reproduction factor (RF) and yield potential. The groups were: tolerant (T) = plant yield well despite of high nematodes attack; moderately tolerant (MT) = plant yield moderately under moderate nematode attack; intolerant (IT) = plants did not yield well even under low nematode pressure; highly tolerant (HT) = plants yield well though under very high nematode attack (Smiley et al., 2014; Dababat et al., 2019).

### Statistical analysis

The CCN data were transformed and then analyzed using analysis of variance (ANOVA). To meet assumptions of normality, Shapiro and Wilk’s (1965) test was conducted. Significant differences between lines were detected using protected least significant difference at *P* < 0.001 using SPSS statistical software V 17.0 (SPSS Inc., Chicago, IL, USA). Linear regression analyses were conducted to describe the relationship between the *H. filipjevi* reproduction factor (RF) and the grain yield for each line in the two experimental areas. Principal component analysis was used to determine population structure using R® 3.4.3 software to distinguish principal groups of wheat lines based on their tolerance to *H. filipjevi*. All other analyses (Grain yield, RF, and yield loss) were described using XLSTAT software 2016.02.28451 (Addinsoft, USA).

## Results

**Figure 1:**
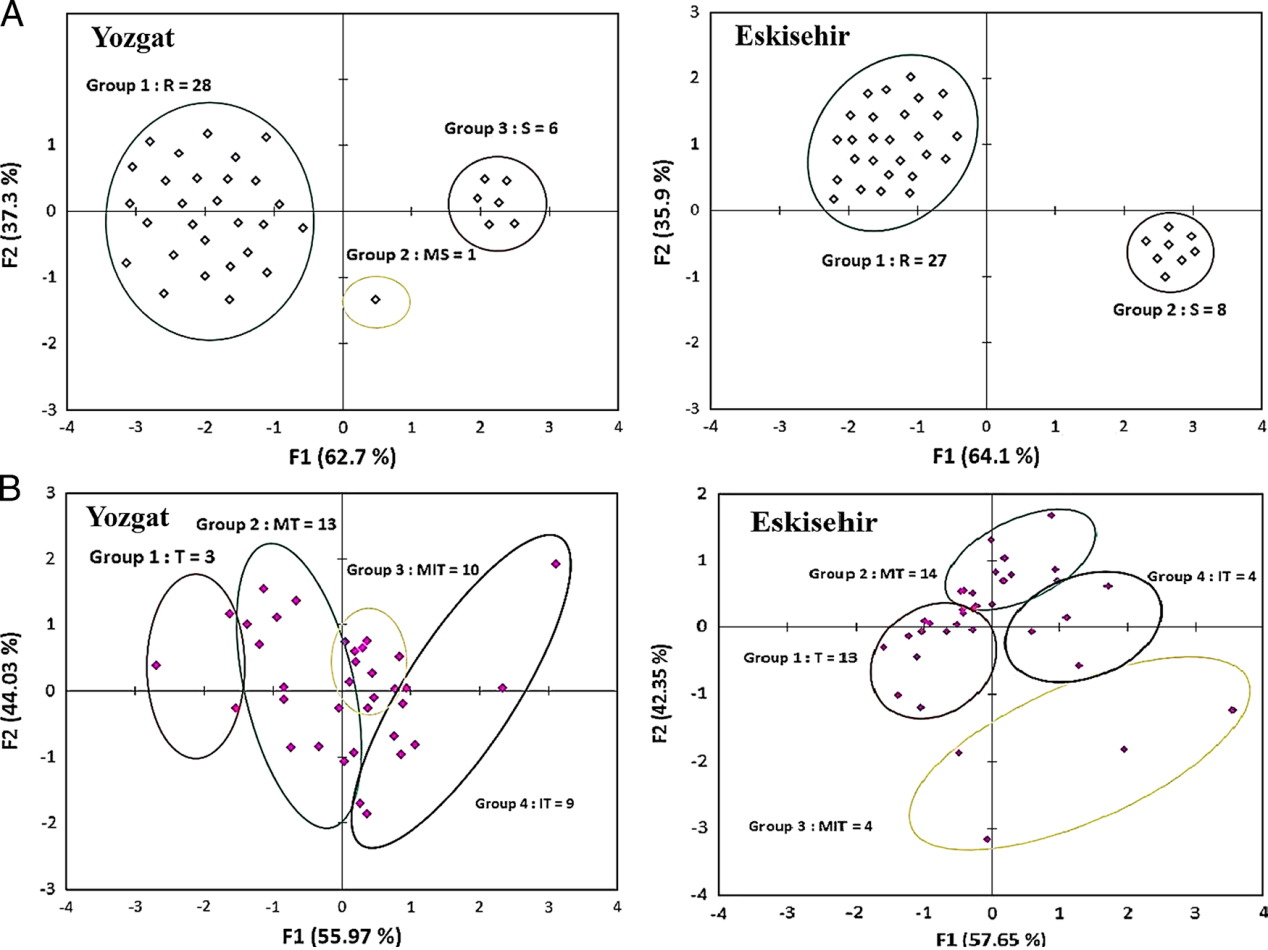
Principal component analysis for the studied 35 lines/cultivars showing the population structure based on their resistance reaction (A) and tolerance reaction (B) in Eskisehir and Yozgat fields conditions.

Results indicated that 23 lines kept their resistant reaction under both field conditions. Thus, only four (L7, L16, L24, and L35) showed susceptibility reaction under both locations (Table 1). Three lines were proven to be tolerant and 13 lines moderately tolerant to *H. filipjevi* population in Yozgat, while 9 and 13 lines were tolerant and moderately tolerant in Eskisehir region, respectively. Around 60% (14 lines) of the resistant lines from TCI were found resistant to both *H. filipjevi* populations including L1, L3, L6, L15, L21, L26, and L34, whereas 17% (four lines) from the USA had the same reaction including L9, L29, L32, and L33. Line 32 gave tolerance and resistance reactions to both *H. filipjevi* populations in both regions. The two susceptible check cultivars “Bezostaya” and “Kutluk” confirmed their susceptibility and intolerance reactions in both locations. Additionally, the two resistant cvs., ‘Katya’ and ‘Sonmez’ conserved their resistance in both locations. Analysis of population structure based on grain (resistance reaction) displayed two distinct groups among the evaluated 35 lines (including checks) in Eskisehir and three distinct groups in Yozgat experiments (Fig. 1A). The same analysis describes the population structure based on obtained tolerance reaction and displayed four distinct groups among the evaluated 35 lines (including checks) in Eskisehir and Yozgat experiments (Fig. 1B).

In the Eskisehir (TZARI) experiment, the first group comprised 27 resistant lines (R), including L12, L13, L14, L22, and L32. Group 2 described eight susceptible line (S), including L12, L16, and L31 (Fig. 1A). With the tolerance reaction, the first group indicated 13 tolerant lines (T), including L6, L12, L19, L20, L21, L28, and L32 as well as L9 (resistant check lines). Group 2 was comprised of 14 moderately tolerant lines (MT), including L2, L5, L10, L24, L30, and L34. Group 3 comprised four moderately intolerant lines (MIT), including L1, L11, and L25. The final group was comprised of four intolerant lines (IT), including L16, L17, L18, and L35 (Fig. 1B). Moreover, L12 in the first group has the best performance as tolerance with grain values (5,250 kg/ha), although the nematode RF was high (2.2). Additionally, the L8 in same group had grain yield values of 4,781 kg/ha and RF of 1.1, whereas L25 in the third group had the lowest grain yield (2,785 kg/ha) with 1.7 RF (Fig. 1). A total of 10 lines acquired with both resistance and tolerance (R, T), including L6, L13, L19, and L21 from TCI, L28 from RUS, and L32 from the USA. Thus, L16 was shown to have susceptible and intolerant reaction (S, IT) alongside with the well-known susceptible check line L35.

In Yozgat (ICAE), we obtained 28 resistant lines (R), including L1, L5, L14, L15, and L32, alongside six susceptible lines (S), including L4, L7, L16, and L20 and one moderately susceptible line (MS), L10 (Fig. 1A), whereas the same analysis displayed four distinct groups in terms of tolerance reaction among the 35 evaluated lines (Fig. 1B). The first group indicated three tolerant lines (T), including L1 and L32. Group 2 comprised 13 moderately tolerant lines (MT), including L5, L14, L22, and L31. Group 3 comprised 10 moderately intolerant lines (MIT), including L2, L3, L8, and L24. The final group comprised nine intolerant lines (IT), including L6, L10, L26, and L33. L32 and L1 in the first group have the best tolerant performance in Yozgat field with grain values of 5187 kg/ha and 3908 kg/ha and RF values of 0.4 and 0.2, respectively. Additionally, the L5 in the second group has a grain value of 3,064 kg/ha and RF of 0.7. L28 in the last group has the lowest grain yield (1,291 kg/ha) although under low nematode population of 0.9 RF, indicating this line was resistant and intolerant (Fig. 1). In this location, only two lines (L1 and L32) were shown to be resistant and tolerant, whereas several lines demonstrated a combination of susceptibility and moderate intolerant reactions including L16, L20, and L24.

As shown in Figure 1, the result from ICEA of lines L7, L12, and L21 showed both higher yields, and higher RF which confirms some tolerance of the lines in the TZARI experiment against *H. filipjevi.* Those lines could be accepted as tolerant lines in the present study. For lines L6, L23, L24, L26, and L29 in ICEA experiment, grain yield was low even at low initial densities of nematodes (*P*_i_), indicating intolerance and susceptibility of those lines to *H. filipjevi.* Line L7 was the only line that combined susceptibility and tolerance to this nematode in both locations, providing a high yield despite high RF.

**Figure 2:**
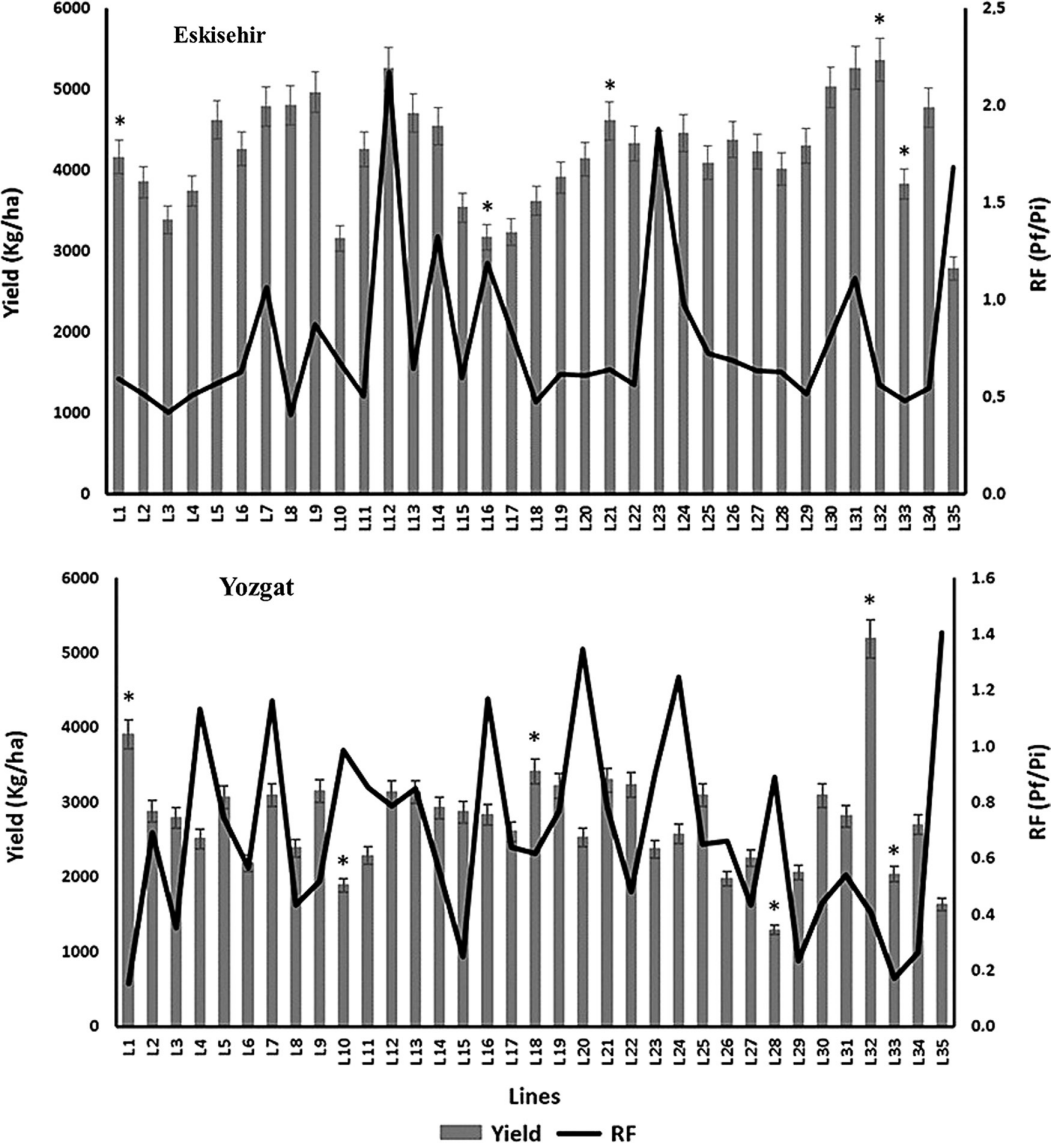
Mean grain yield and *Heterodera filipjevi* reproduction factor (RF) in both experiments during the 2014 to 2015 and 2015 to 2016 growing seasons. Stars represent homogeneous groups based on protected least significant difference test for each variable at *P* < 0.001. Error lines on the bars represent the standard error (*n* = 6).

We detected significant differences (*P* < 0.001) in the host status of the 31 IWWIP lines tested to *H. filipjevi* based on their yield and reproduction factor (RF) in field conditions in TZARI (RF) (Fig. 2). The highest yield was obtained from the L32 (5,363 Kg/ha), which showed tolerant and resistant reactions to both nematode populations. On the other hand, the L35 (check line) gave the lowest yield (2,785 Kg/ha) with an intolerant reaction. Of the tested 31 lines, 5 lines (L4, L11, L29, L33, and L34) were as resistant as the controls with known resistance to *H. filipjevi* (L27) and significantly lower RF compared to the other IWWIP lines evaluated in this study. The lowest RF was achieved by L3 (0.4), while L12 gave the highest RF in the Eskisehir field experiment (2.2) (Fig. 2).

The results indicated that there was a significant difference (*P* < 0.001) in the host status of the IWWIP lines to *H. filipjevi*, ranging from resistant to susceptible in terms of the reproduction factor, which fluctuated from 0.2 to 1.4 in ICEA field (Fig. 2). Among the studied lines, four (L5, L3, L15, and L29) were as resistant as the controls with known resistance to *H. filipjevi* (L18 and L27) and significantly lower RF compared to the other IWWIP lines evaluated in this study. The lowest RF was achieved by L1 (0.2), while both L20 and L35 gave the highest RF values (1.3 and 1.4, respectively). A total of 27 lines were shown to maintain resistance in Yozgat region (Fig. 2). The highest yield was obtained by L32 (5,187 kg/ha), indicating that this line is both resistant and tolerant to *H. filipjevi.* While the lowest yield was obtained by L28 (1,291 Kg/ha) with a resistance capacity and intolerant reaction even with a RF value of 0.9 (Fig. 2).

**Figure 3:**
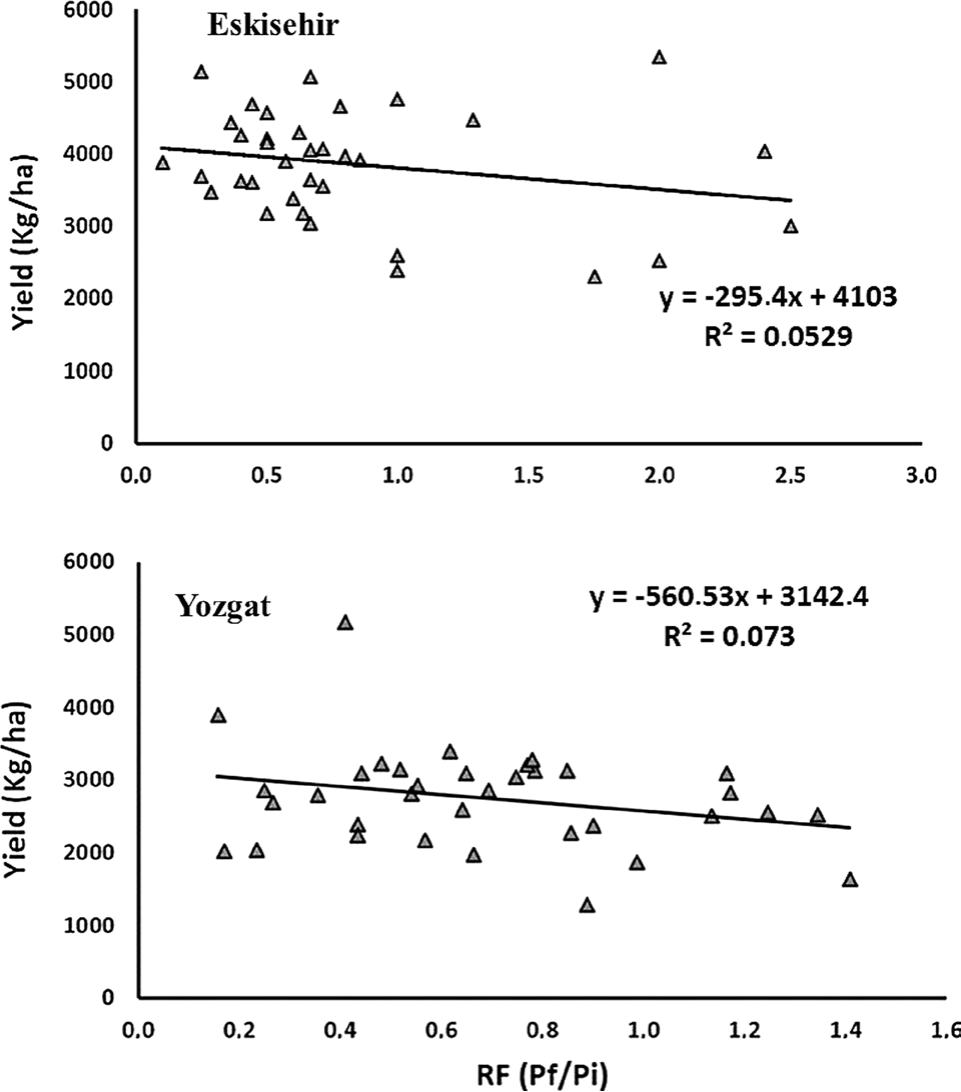
Log-linear regressions of grain yield and *Heterodera filipjevi* reproduction factor (RF) in Eskisehir and Yozgat experiments. All *R* ^2^ values were significant at *P <* 0.05. Values are means of two years, each with three replicates. The link represents the predicted linear regression model. Equations were represented on kg ha^−1^.

To assess further association between grain yield and nematodes RF values, linear regression analysis was used. In both regions (Eskisehir and Yozgat), we found a negative relationship between yield and RF of *H. filipjevi* (Fig. 3), which describes the negative impact of this nematode on winter wheat germplasm studied. In Eskisehir, the lines L1, L3, L8, L9, L15, L22, L29, and L32 were the most resistant, with RF values of 0.6, 0.4, 0.4, 0.9, 0.6, 0.6, 0.5, and 0.6, respectively (Fig. 3). Reference lines L16, L18, L27, and L35 had RF values of 1.2, 0.5, 0.6, and 1.7, respectively. Regression analyses clearly showed that *H. filipjevi* RF was negatively related with grain yield in Yozgat experiment (Fig. 3). Our result showed that high *H. filipjevi* populations were associated with yield reduction of the susceptible winter wheat lines, where lines L4, L7, L23, and L24 showed higher yield reduction under reproduction factor values of 1.1, 1.2, 0.9, and 1.2, respectively (Fig. 3).

## Discussion

The current study reports the first quantifiable and comprehensive evidence of the host status of different winter wheat accessions under *H. filipjevi* infested fields in the provinces of Yozgat and Eskisehir in Turkey. The results of this study indicate that *H. filipjevi* is considered an important factor which causes severe damage to different wheat cultivars and has potential to decrease growth and yield of wheat in Turkey (Dababat et al., 2015; Toktay et al., 2015; Imren et al., 2016, 2017; Yıldız et al., 2017). The resistance/tolerance reactions of the 31 winter wheat lines evaluated in this study were identified with a range of responses, from resistant to susceptible and tolerant to intolerant to *H. filipjevi*.

Resistant or tolerant wheat cultivars are the most successful and preferable method to manage nematodes in cereals (Dababat et al., 2014). Tolerant varieties suffer from the infection with little yield reduction even when their roots are invaded by nematodes, while resistant varieties reduce the rate of nematode multiplication in the roots (Roberts, 2002). Therefore, resistance should be combined with tolerance, the ability of the host plant to maintain yield potential in the presence of the nematode (Cook and Rivoal, 1998). Moreover, wheat varieties with resistance or tolerance have been shown to provide resistance against a wide variety of both biotic and abiotic stresses (Kimber and Feldman, 1987). Wheat yield depends on the interaction between ecological and edaphic factors, but it is highly responsive to *Heterodera* species infection, even when other stresses restrict yield (Smiley et al., 2005). Therefore, the use of resistant or tolerant lines requires a sound knowledge of the virulence spectrum and pathotypes of the targeted species of nematode (Majnik et al., 2003; Smiley et al., 2005). As a result, wheat accessions resistant or tolerant to *H. filipjevi* populations in one region might be fully susceptible to populations in other regions (Smiley et al., 2005). Findings of this study indicated that around 23 lines kept their resistant reaction under field conditions; three lines were proven tolerant and 13 lines moderately tolerant to *H. filipjevi* (Yozgat), while 13 lines were tolerant, and 14 lines were moderately tolerant in Eskisehir region. This location indicated four intolerant lines, fewer in comparison to Yozgat region, as it comprised nine intolerant lines, which can explain the reduction of tolerance can be caused by high nematodes densities.

Nowadays, wheat landraces and domesticated wheat have been used to identify many important agronomical traits (Kimber and Feldman, 1987; Van-Slageren, 1994). Although considerable research has been performed to identify resistance sources in wheat, no durable resistant cultivar to *Heterodera* species is currently available. Therefore, there is an urgent need to identify new resistance sources and to pyramid and incorporate them into high-yielding cultivars. Many studies have attempted to seek new genotypes of cyst nematode resistance (Smiley et al., 2005; Hajihasani et al., 2010; Fard et al., 2018). Nematode resistance or tolerance in wheat has been reported from pot (glasshouse and growth chamber) and field experiments (Hajihasani et al., 2010; Şahin et al., 2010; Moustafa et al., 2018). Hajihasani et al. (2010) reported that several growth parameters (plant height, root dry weight, aerial shoot dry weight, and grain yield) of the wheat cultivar “Sardari” were significantly decreased when *H. ﬁlipjevi* populations increased. Their results showed that *H. filipjevi* caused significant reduction in grain yield when grown under high nematode initial population density. Yuan et al. (2011) investigated the resistance of 75 wheat cultivars or lines from CIMMYT under greenhouse and field conditions using a relative resistance index (RRI) and *P*_f_/*P*_i_ ratios of *H. filipjevi* population from Xuchang, Henan province, China and found no cultivar was immune to *H. filipjevi*. However, a few entries displayed resistance or high resistance in both environments, such as 6R(6D), MACKELLER, CPI 133842, CPI 133814, and CROC_1/AE.SQUARROSA (224) // OPATA*1. Moustafa et al. (2018) screened 17 local and international wheat genotypes against Hal population of *H. avenae* in Saudi Arabia and their results showed that the 10 studied wheat genotypes were significantly different in terms of their resistance and tolerance to nematode. On the other hand, CIMMYT spring wheat genotypes (15 SAWYT-30, 15 SAWYT-31, 15 SAWYT-38, and 15 SAWYT-42) plus the cvs. AUS-30851 and Yecora Rojo were found to be the most susceptible genotypes to the tested Saudi population of *H. avenae*. Fard et al. (2018) reported that grain yield was significantly affected by *H. filipjevi* as well as other growth parameters in all Back–cross Rowshan, Pishtaz, and Parsi cultivars in the province of Isfahan in Iran.

In Turkey, breeders have selected wheat varieties for *Heterodera* resistance for decades due to high infection pressure. Şahin et al. (2010) also screened 150 international and national wheat varieties against *H. filipjevi* under *in vitro* conditions and found that 5 international (Hartog, Katea, HN7/OROFEN/BJN8/3/SERI, IWA8604765, and IWA8608077) and 10 national wheat genotypes (Yakar 99, Sönmez, Kırmızı Mısri, Altındane 12, Kunduru 1149, Yelken 2000, Üveyik, Sorgül, Germir, and Tosunbey) were the most resistant wheat variety and pedigrees found against *H. filipjevi* Haymana population. Imren et al. (2012) investigated 82 wheat genotypes for their responses to *H. avenae* under *in vitro* conditions. They reported that 4 national varieties (including Adana 99 (PFAU/SERI82//BOG“S”), 23 international germplasm, and 17 wild genotypes were found moderately resistant to nematode. Toktay et al. (2012) screened 42 CIMMYT wheat lines originating from a cross between the Middle Eastern resistant landrace ‘AUS4930 7.2’ and the widely adapted high yielding but susceptible CIMMYT line ‘Pastor’ and found that 5 and 9 of these lines were resistant and moderately resistant to the Haymana population of *H. filipjevi*, respectively. Imren et al. (2015) investigated six common bread wheat varieties grown in Turkey for their responses to *H. avenae* under field conditions. They found no varieties with complete resistance to *H. avenae*. However, they reported Adana 99, Ceyhan 99, and Silverstar were moderately resistant and that Karatopak, Osmaniyem, and Seri 82 were moderately susceptible against *H. avenae*. Yavuzaslanoglu et al. (2016) reported that among the screened Iranian wheat landrace accessions against *H*. *filipjevi,* one germplasm [PI628144 (syn. AUS28321)] was resistant and five other accessions were moderately resistant under greenhouse conditions. Dababat et al. (2019) reported 484 of CIMMYT’s spring wheat accessions for resistance to *P. thornei* of which 56 lines were pre-identified as resistant under controlled growth room conditions. These lines were further evaluated for their resistance and tolerance reactions under field conditions, where 14 accessions maintained their resistance, and 16 were moderately resistant against *P. thornei*. Four lines gave excellent resistant and tolerance reactions to *P. thornei*. The relationship between the nematode reproduction factor (*P*_f_/*P*_i_) and wheat grain yield in field experiments fit a linear regression model. In this study, the reaction of the 31 lines plus the four checks showed that around 10 lines were resistant and tolerant in Eskisehir, whereas just two lines gave the same reaction in Yozgat. The line ‘L32’ provided resistant reaction and tolerance capacity to both *H. filipjevi* populations.

The low reproduction factor of the tested lines is normal, as those lines were screened many times under the controlled conditions for *H. filipjevi* as they were resistant against it. The RF normally goes to a value that exceeds six in nurseries screened for the first time. Our results indicated significant negative relationship between RF and wheat yields. The reduction in grain yield increased with both increasing *P*_i_ and *P*_f_. Similar results reported by Imren and Elekcioğlu (2014) who described a negative relationship between the RF of *H. avenae* and grain yield of three cultivars Seri 82, Osmaniyem, and Karatopak in the Eastern Mediterranean region of Turkey. The negative relationship between nematode *P*_i_ densities and grain yields for the tested germplasm in Eskisehir are similar to those of microplot trials conducted in Iran on Sardari cultivar by Hajihasani et al. (2010) and Fard et al. (2018) and to the field experiments conducted by Smiley et al. (2005).

Wheat germplasm with acceptable levels of resistance and tolerance are subsequently crossed with high-yielding susceptible cultivars. This is an essential step as most of the locally adapted wheat varieties are susceptible to *H. filipjevi* and identifying a new resistant wheat germplasm will allow breeders to develop new crosses with their local varieties, improving their genetic resistance to the targeted nematode species. So far, hundreds of CCN-resistant genotypes have been identified from the IWWIP materials, but the genetic nature of the resistance and genetic diversity of these germplasms are not yet known. Therefore, studying the genetic background of those lines is important to understand the resistance novelty of those lines and use those lines with different genetic backgrounds in crosses and for pyramiding a new resistance and high yield that increases grain yield to support food security.
